# Barriers to and facilitators of adherence to evidence-based standard antimicrobial treatment guidelines among physicians in Ethiopia: a formative qualitative study

**DOI:** 10.1038/s41598-026-41472-9

**Published:** 2026-02-27

**Authors:** Minyahil Tadesse Boltena, Mirkuzie Woldie, Ziad El-Khatib, Yibeltal Siraneh, Abraham Sahilemichael Kebede, Zelalem Tadesse, Wondwossen Amanuel, Sudhakar Morankar

**Affiliations:** 1 Ethiopian Evidence Based Health Care Centre: A Joanna Briggs Institute Center of Excellence, Health Behavior and Society Department, Faculty of Public Health, Institute of Health, Jimma University, Jimma, Ethiopia; 2 Artificial Intelligence and Digital Health Innovation Lab, Armauer Hansen Research Institute, Ministry of Health, Addis Ababa, Ethiopia; 3 Global Public Health Department, Karolinska Institute, Solna, Sweden; 4 Lero SFI Research Centre for Software, Health Research Institute, University of Limerick, Limerick, Ireland; 5 Africa Centers for Diseases Control and Prevention (Africa CDC), Addis Ababa, Ethiopia; 6 Ministry of Health, Addis Ababa, Ethiopia; 7 Health Policy and Management Departments, Faculty of Public Health, Institute of Health, Jimma University, Jimma, Ethiopia; 8 Fenot Project, Fenot Associates Plc, Addis Ababa, Ethiopia

**Keywords:** Antimicrobial stewardship, COM-B model, Ethiopia, Physicians’ adherence, Qualitative study, Rational antimicrobial prescription, Standard treatment guidelines (STGs), Health care, Medical research

## Abstract

Rational antimicrobial prescribing is critical to curb antimicrobial resistance, yet adherence to standard antimicrobial treatment guidelines remains inconsistent in Ethiopia. Understanding the barriers and facilitators influencing physicians’ adherence is essential to inform context-appropriate interventions. We conducted a formative qualitative study from April to June 2023 in 20 public hospitals across four Ethiopian regions. Using maximum variation purposive sampling, 47 physicians participated in in-depth interviews. Data were analyzed using the Theoretical Domains Framework embedded within the COM-B model, with thematic coding in MAXQDA and reporting guided by COREQ. Physicians faced multiple barriers to standard antimicrobial treatment guidelines adherence. The guidelines were often bulky, outdated, and misaligned with patient needs. Limited training, low confidence, and resistance from senior clinicians further reduced adherence. Delayed dissemination, restricted access to medicines, heavy clinical workloads, and inconsistent practices in private facilities compounded these challenges. Facilitators supporting adherence included peer collaboration, routine clinical audits, trust in locally developed guidelines, and professional motivation. Study participants recommended developing smartphone application of standard antimicrobial treatment guidelines for point-of-care access. Study participants highlighted digital solutions, such as mobile STG applications, to enhance accessibility and recommended interventions including training, digital access, audits, and policy enforcement. Adherence to standard treatment guidelines is influenced by individual, institutional, and system-level factors. Updating and digitalizing guidelines, integrating adherence into continuous professional development training, strengthening institutional support, and implementing policy measures are key strategies to improve adherence and rational antimicrobial prescription.

**Supplementary Information:**

The online version contains supplementary material available at 10.1038/s41598-026-41472-9.

## Background

Antimicrobial resistance (AMR) is a critical global health threat that could reverse decades of medical progress^1^. Global projections, largely based on data from high-income countries, estimate up to 10 million annual deaths by 2050 due to AMR^2^. However, these estimate may underrepresent the burden in low- and middle-income countries because of weaker surveillance systems, limited diagnostic capacity, and unregulated antibiotic use^3^. In this context, evidence-based standard antimicrobial treatment guidelines (STGs) are essential tools for promoting rational prescribing and antimicrobial stewardship, yet adherence remains suboptimal^4^. Guidelines provide context-appropriate recommendations on diagnosis, drug selection, and treatment duration^5^. When implemented, they standardize care and reduce inappropriate prescriptions, which is particularly important in low-income countries where AMR burden is high and stewardship resources are limited^6^.

In sub-Saharan Africa (SSA), barriers to STGs adherence includes limited access to updated guidelines, frequent drug shortages, inadequate diagnostic services, and high patient load^7^. These constraints often compel physicians to rely on empirical treatment rather than guideline recommended care. While several quantitative studies have measured adherence levels and prescribing patterns in the region, in-depth qualitative understanding of the physicians’ experiences, decision-making processes, and contextual influence remains limited. As a result, many stewardship interventions lack a strong formative evidence base tailored to local clinical realities.

Ethiopia faces alarming AMR trends, with resistance rates for pathogens such as *Klebsiella pneumoniae* and *Staphylococcus aureus* exceeding 60%^8^. To promote evidence-based antimicrobial prescribing practice, The Ministry of Health in Ethiopia introduced STGs in 2014, updated it in 2021^9^. Despite these efforts, adherence to STGs remain poor; nearly half of outpatient antimicrobial prescriptions were non-compliant with STGs in tertiary hospitals^10^. Systemic barriers including weak dissemination of guidelines, inadequate laboratory infrastructure, fragmented referral pathways, and inconsistent drug supply, continue to undermine rational antimicrobial prescribing and accelerate resistance^11^.

Irrational antimicrobial use in private healthcare facilities, including routine prescription of broad-spectrum agents without microbiological evidence further complicates adherence when patients are referred to public hospitals^12^. Although prior studies have reported lower adherence to STGs, only a small number of published qualitative studies have systematically explored the underlying barriers and facilitators across public hospitals and professionals, including general practitioners, residents, and specialists in Ethiopia^13,14^. Consequently, critical issues such as the accessibility and physicians trust in STGs, and the influence of the private health sector prescribing practices remains insufficiently understood^15^.

To address these gaps, this study is embedded within a type I effectiveness-implementation cluster randomized trial. It seeks to generate contextualized insights to improve STGs adherence by exploring physicians’ experiences across public hospitals in Ethiopia. By identifying the barriers, facilitators, and practical solutions, the findings will inform targeted strategies to strengthen rational antimicrobial prescribing and support the implementation of National AMR Action Plan in Ethiopia.

## Methods

### Study setting and context

This formative qualitative study was conducted between April and June 2023 across selected public hospitals in four administrative regions of Ethiopia: Addis Ababa, Amhara, Central Ethiopia, and Oromia. The study sites were purposefully selected to capture a range of public hospital types, geographic locations, and healthcare delivery contexts. To ensure diversity at the health system level, public hospitals, including primary, general, and specialized/referral, were selected via purposive sampling^16^. Public primary hospitals, which are commonly located in rural and underserved areas, provide essential inpatient and outpatient services^16^. Public general hospitals offer a broader range of services, including surgery and specialized outpatient care. Public specialized or referral hospitals, typically located in urban centers, manage complex cases requiring advanced diagnostics and multidisciplinary care. This structured selection approach aimed to provide rich contextual variation to understand physician experiences and decision-making regarding adherence to evidence-based STGs across different settings.

### Study design

An exploratory descriptive qualitative design was employed to inform the formative assessment, which is guided by the theoretical domain framework (TDF) embedded within the COM-B model. The design was selected to capture a wide range of experiences and perspectives from diverse clinical settings, enabling a nuanced understanding of both individual and systemic factors contributing to rational or irrational antimicrobial prescription and use^17^. The approach enabled structured inquiry into the capabilities, opportunities, and motivations that shape antimicrobial prescription behavior. Given that compliance with evidence-based clinical practices, particularly antimicrobial prescribing, requires coordinated effort across professional, institutional, and policy levels, this approach allows the identification of key enablers and constraints across these domains.

### Study participants

The study participants consisted of health professionals directly involved in prescribing antimicrobials, including general practitioners, resident physicians, and specialist doctors such as internists, pediatricians, surgeons, obstetricians/gynecologists, and critical care specialists. These participants were selected via a maximum variation purposive sampling strategy to ensure a diverse professional mix that reflects the range of prescribing practices across different departments and levels of care^18^. Given the study’s focus on exploring adherence to evidence-based STGs, participants were intentionally drawn from clinical roles where prescribing decisions are routinely made. This approach enables the exploration of various experiences related to rational and irrational antimicrobial prescription and use, perceptions of evidence-based clinical practice, and the contextual barriers and enablers that influence compliance with STGs in real-world public hospital settings^19^. While purposive sampling across four regions enabled contextual depth and variation, the findings are not intended to be statistically generalizable beyond similar public hospital settings.

### Theoretical framework

The COM-B model (Capability, Opportunity, Motivation – Behavior) was employed as the overarching framework to guide both data collection and analysis. This model was selected because it provides a comprehensive and practical lens for understanding the multifactorial drivers of clinician behavior, particularly in complex health system settings involving antimicrobial prescribing in Ethiopia^20^. Unlike frameworks that emphasize only individual knowledge or attitudes, COM-B explicitly integrates individual, social, and structural influences, making it well suited to capture the interplay between personal competencies, contextual realities, and motivational dynamics that shape adherence to STGs^21^.

**Fig. 1:**
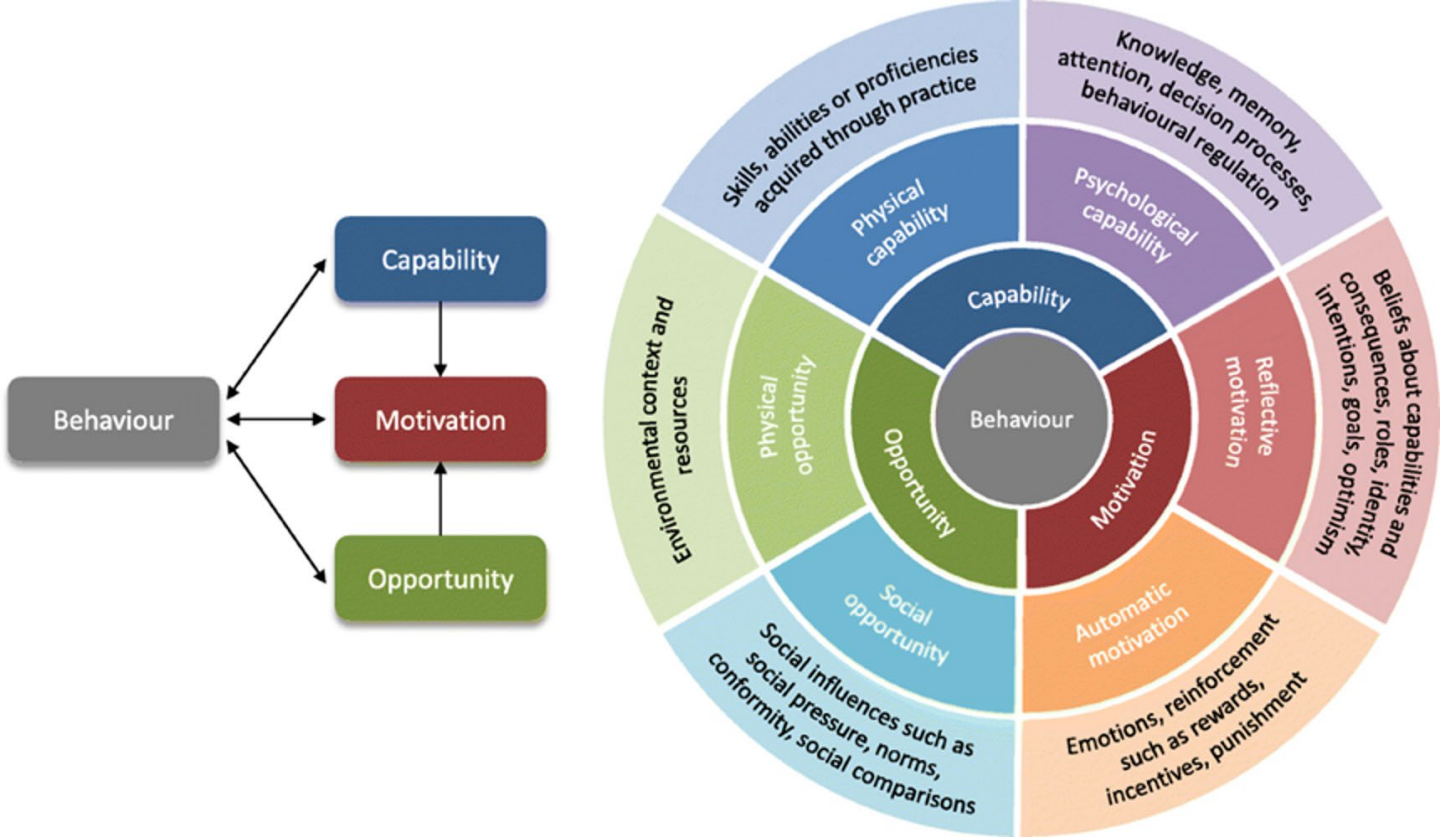
The TDF embedded within the COM-B model to explore the barriers and facilitators of adherence to evidence-based STGs among physicians in Ethiopia.

We embedded the theoretical domains framework (TDF) within COM-B to deepen our exploration of behavioral determinants^22^. The TDF expands each COM-B component into 14 detailed domains, allowing us to systematically examine areas such as knowledge, professional role and identity, the environmental context, beliefs about consequences, and memory/attention processes^23^. This combined use of COM-B and TDF enhanced both the robustness and interpretability of the qualitative analysis, ensuring that our findings could be directly mapped to evidence-informed strategies for behavior change^24^. By applying COM-B with the embedded TDF, we were able to move beyond simply identifying barriers and facilitators. We generated a structured understanding of why physicians may or may not adhere to STGs and which specific levers could be targeted to promote behavior change. This approach aligns well with the objectives of our type I effectiveness-implementation cluster randomized controlled trial, in which these qualitative findings will directly inform the design, tailoring, and refinement of implementation strategies aimed at improving STGs-concordant prescribing (Fig. 1)^25^.

### Sample size and sampling technique

Public hospitals were chosen to reflect a mix of primary, general, and specialized care facilities, ensuring variation in patient load, clinical capacity, and antimicrobial prescribing practices. Within each public hospital, physicians, including general practitioners, resident doctors, and specialists, were selected on the basis of their direct involvement in antimicrobial prescription. Predefined criteria guided the selection, focusing on professional role, years of experience, and departmental representation to ensure comprehensive insights into barriers to and facilitators of adherence to evidence-based STGs.

The participants were identified in collaboration with public hospital administrators and department heads to ensure relevance and representation across major clinical units such as internal medicine, pediatrics, surgery, obstetrics/gynecology, and emergency care. The identification process involved department heads reviewing clinical duty rosters to pinpoint physicians with the highest frequency of antibiotic prescribing responsibilities. These individuals were then purposively approached to ensure the study participants included a strategic mix of general practitioners,  junior residents, and senior specialists. This structured identification ensured that the study participants possessed the specific clinical experience necessary to discuss the systemic barriers to STGs adherence within the Ethiopian healthcare context. The study participants had between 2 and 23 years of clinical experience, providing a rich range of perspectives on rational and irrational antimicrobial prescription, compliance with evidence-based STGs, and systemic challenges influencing evidence-based clinical practice. The final sample size was determined by data saturation using an iterative approach to data collection and analysis. After each interview, preliminary coding was conducted to monitor the emergence of new concepts related to STG adherence. Saturation was defined as the point of code stability, when three consecutive transcripts yielded no new codes and existing categories were sufficiently developed to explain prescribing behaviors. Saturation was achieved after 42 interviews; five additional interviews were conducted to confirm consistency across hospital types, resulting in a final sample of 47 physicians.

### Data collection tool and procedure

Data were collected through in-depth interviews (IDIs) with 47 physicians actively involved in antimicrobial prescribing, including general practitioners, resident physicians, and specialists such as internists, pediatricians, surgeons, obstetricians/gynecologists, and critical care experts and department heads, via an interview guide specifically developed to explore the barriers and facilitators influencing physicians’ adherence to evidence-based STGs (supplementary file 1). We conducted in-depth interviews (IDIs) via an interview guide initially developed in English and translated into Amharic and Afan Oromo to ensure clarity and linguistic inclusivity across regions. The interview guide was translated from English into Amharic and Afan Oromo and back-translated into English by independent bilingual experts to ensure semantic equivalence and conceptual consistency. Data collection was carried out by trained qualitative researchers with master’s-level qualifications who were recruited from universities within each study region. These data collectors were experienced in conducting in-depth interviews in clinical and health systems research.

The guide was tailored to investigate key issues, including perceptions of STGs relevance, sources of clinical decision support, departmental and institutional prescribing norms, barriers to rational antimicrobial prescription, and enablers of compliance with evidence-based clinical practice. Additional questions asked about challenges related to irrational antimicrobial use, awareness of antimicrobial resistance containment policies, and strategies for improving STGs adherence in routine public hospital care. All interviews were conducted in the participants’ usual work settings within the public hospitals, such as consultation rooms or departmental offices, to ensure comfort, privacy, and contextual relevance. The interviews were audio-recorded with participant consent and complemented by detailed field notes, which captured nonverbal cues and contextual observations.

Each IDI lasted approximately 30 to 60 min. The field team ensured that discussions remained participant driven, allowing physicians to express their views freely and provide detailed reflections on real-life prescribing practices, institutional influences, and perceived gaps in evidence-informed antimicrobial stewardship.

### Rigor and trustworthiness of the study

To ensure the rigor and trustworthiness of this qualitative investigation, we adhered to the four foundational principles of qualitative research: credibility, dependability, confirmability, and transferability^26^. These principles guided both data collection and analysis, ensuring the reliability and validity of findings related to physicians’ adherence to evidence-based antimicrobial treatment STGs.

Prior to data collection, the semi-structured interview guide was pretested with a small sample of physicians from public hospitals not included in the study, allowing for refinement on the basis of clarity, relevance, and flow. Data collectors received comprehensive training not only on the technical aspects of conducting in-depth interviews but also on the study’s core focus, rational versus irrational antimicrobial prescription practices, and compliance with evidence-informed antimicrobial resistance (AMR) containment policies. To enhance credibility, experienced researchers involved in the data collection also led transcription and preliminary analysis, allowing them to contextualize spoken responses with observed nonverbal cues, emotional tones, and interpersonal dynamics. This continuity enabled a richer interpretation of physicians’ reflections on barriers and enablers to evidence-based clinical practice.

The inclusion of a professionally and regionally diverse group of participants (i.e., from different public hospital levels, specialties, and geographic regions) strengthened data triangulation, allowing for a broader understanding of contextual and systemic influences on antimicrobial prescribing. To reinforce dependability and confirmability, an audit trail was maintained, including all audio recordings, corresponding transcripts, and analytic memos. Member checking was conducted during follow-up discussions and feedback sessions, where preliminary findings were validated and refined on the basis of participants’ input. These validation activities, carried out during team meetings and stakeholder workshops, further enhanced the trustworthiness of the study and its potential to inform targeted, context-sensitive interventions.

### Data management and analysis

All audio recordings from the in-depth interviews were transcribed verbatim into English by the same experienced researchers who conducted the interviews. This ensured continuity and preserved the contextual nuances of physicians’ responses related to adherence to evidence-based STGs and the dynamics of rational versus irrational antimicrobial prescription.

Following an initial close reading of all the transcripts and reflection on the interview guide, a comprehensive codebook was developed collaboratively by the research team. The codebook included each code, its definition, representative quotes from the transcripts, and the subthemes and overarching themes to which the code belonged (supplementary file 2). Both researchers independently coded all transcripts using the finalized codebook. The initial inter-rater agreement was 78%, indicating substantial consistency. Any discrepancies in coding were resolved through discussion and consensus, with reference to the transcripts and code definitions, ensuring reliability and rigor in the thematic analysis. Coding was conducted via an abductive thematic analysis approach in MAXQDA (version 2020) by two researchers^27^. Thus, we began with verbatim transcription, and many codes were generated. These codes were then grouped into subthemes, which were further thematized into main themes. Finally, the main themes were later packaged into the categories of the theoretical frameworks and their constructs (COM-B).

The codebook served as a guiding framework throughout the analysis and was used in organizing and presenting the results. Themes were reported with thick, descriptive analysis and supported by direct quotes from participants to ensure the authenticity and depth of their voices. All methods and analytical procedures are documented and reported in line with the Consolidated Criteria for Reporting Qualitative Research (COREQ) to ensure transparency and reproducibility^28^(supplementary file 3).

### Ethical considerations

The ethical approval for this study was obtained from the Armauer Hansen Research Institute Institutional Ethics Review Committee with reference number PO-062-22. This study is part of an implementation research PhD project at Jimma University, Institute of Health, with IRB approval code JUIH/IRB/235/25 (Supplementary file 4). All participants received detailed information about the study’s purpose, objectives, potential risks, and benefits, and provided written informed consent. They were explicitly informed of their right to withdraw at any time without consequences. Confidentiality and privacy were strictly maintained, with all data securely stored and accessible only to the research team, ensuring a trustworthy environment for participants to discuss STG adherence and antimicrobial prescribing practices. 

## Results

### Characteristics of the study participants

The study was conducted across 20 public hospitals selected from four regions of Ethiopia: Addis Ababa, Amhara, Oromia, and Central Ethiopia. The public hospital selection included a mix of facility types reflective of Ethiopia’s tiered health system: 12 public general hospitals and an equal number of four public specialized referral hospitals and four public primary hospitals.

A total of 47 physicians participated in the study. Most were male 35 (74.5%), whereas 12(25.5%) were female. In terms of specialty, 11(23.4%) were general practitioners, 9(19.1%) were infectious disease specialists, 7(14.9%) were obstetricians/gynecologists, 6(12.8%) were pediatricians, 5(10.6%) were internal medicine physicians, 5(10.6%) were infection prevention and control specialists, and 4(8.5%) were surgeons. With respect to years of clinical work experience in antibiotic prescription, 17(36.2%) had 1–3 years, 14(29.8%) had 4–6 years, 10(21.3%) had 7–9 years, and 6(12.8%) had ≥ 10 years of experience. More than half of the participants worked in public general hospitals 26 (55.3%), followed by public comprehensive referral hospitals 12 (25.5%) and public primary hospitals 9 (19.1%). Regionally, 15 (31.9%) were from Oromia, 13(27.7%) were from Addis Ababa, 10(21.3%) were from Central Ethiopia, and 9(19.1%) were from Amhara (Table 1).

**Table 1:** Socio-demographic characteristics of study participants (*N* = 47).

Variable	Category	Frequency (*n*)	Percentage (%)
Gender	Male	35	74.5
	Female	12	25.5
Education and medical specialty	General practitioner	11	23.4
	Internal medicine	5	10.6
	Pediatrics	6	12.8
	Infectious diseases	9	19.1
	Infection prevention & control	5	10.6
	Surgery	4	8.5
	Obstetrics & gynecology	7	14.9
Years of clinical work experience	1–3 years	17	36.2
	4–6 years	14	29.8
	7–9 years	10	21.3
	≥ 10 years	6	12.8
Type of public hospital setting	General	26	55.3
	Primary	9	19.1
	Comprehensive referral	12	25.5
Region	Addis Ababa	13	27.7
	Amhara	9	19.1
	Oromia	15	31.9
	Central Ethiopia	10	21.3

### Mapping findings to the COM-B framework

**Fig. 2:**
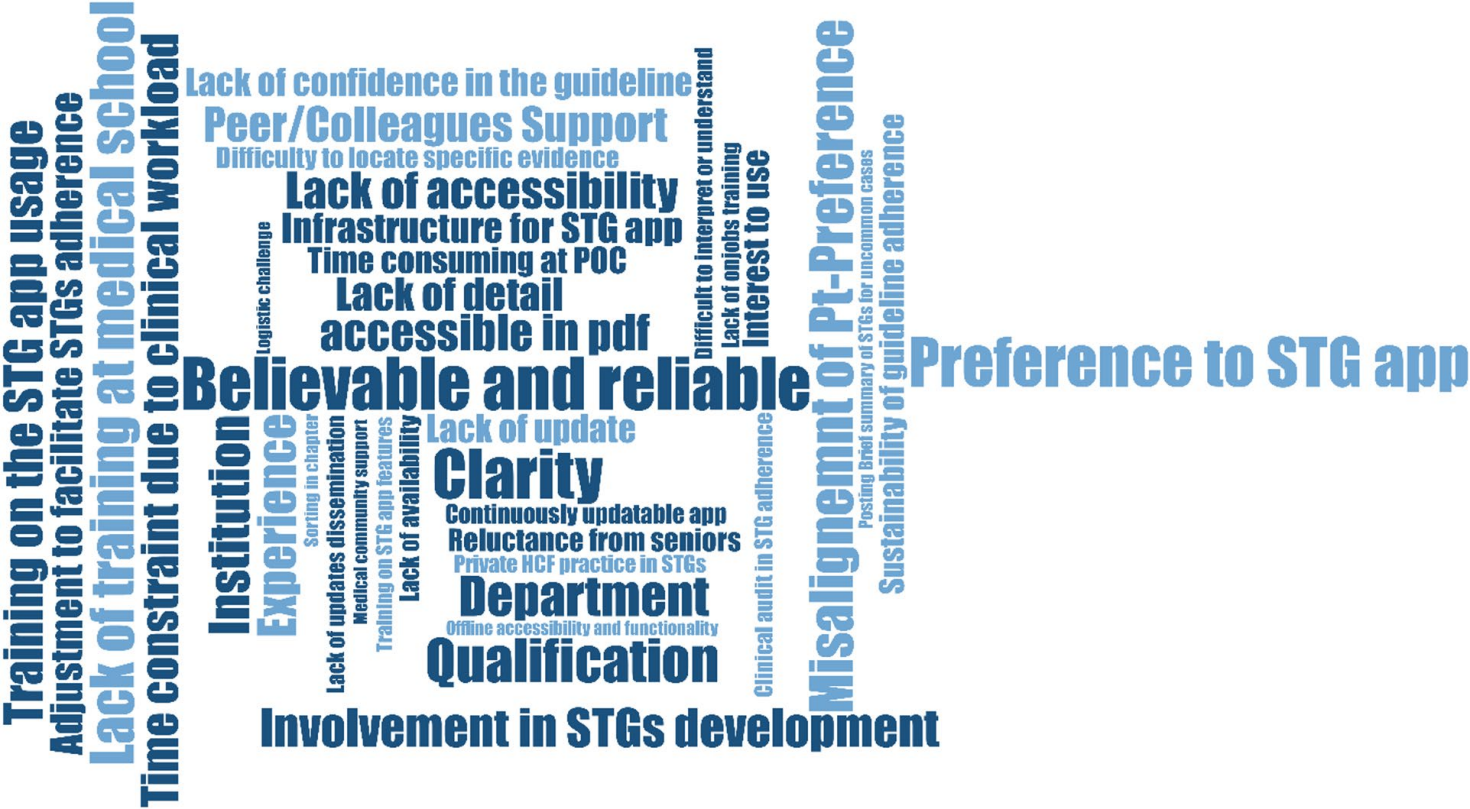
Packed cloud visualization of the barriers and facilitators showing frequency occurrence of codes emerged (MAXQDA output).

Following transcription and review of all 47 interviews, the team generated over 410 initial codes capturing insights into clinical decision-making and systemic influences. These were organized into eight subthemes and consolidated into five overarching themes: perceptions of evidence-based prescribing, personal experiences with irrational antimicrobial use, barriers to and facilitators of STGs adherence, and suggested institutional and policy-level interventions. Each theme and sub-theme is supported by relevant quotes from the transcripts (Fig. 2). The main findings are reported as barriers, facilitators, and recommended implementation adaptation strategies (Table 2). Physicians valued STGs for guiding rational antimicrobial use but faced gaps in training, outdated content, and usability challenges.

This packed cloud illustrates the frequency and prominence of key barriers and facilitators influencing adherence to evidence-based STGs. Each word or phrase represents a code or subtheme identified from the qualitative interviews, with larger text indicating higher prevalence among participants. Dominant themes include “Believable and reliable,” “Clarity,” “Preference to STG app,” “Lack of accessibility,” and “Involvement in STG development,” highlighting critical areas of capability, opportunity, and motivation that shape STGs adherence. Beyond showing frequency, the cloud highlights how facilitators such as peer support, training on STG app usage, and active participation in guideline development strengthen physician capability and motivation, while contextual barriers including patient preference misalignment, limited offline access, and high clinical workload constrain opportunity. The prominence of “Preference to STG app” underscores the importance of digital implementation strategies, such as mobile STG applications, to improve accessibility, usability, and adoption. The relative size of terms emphasizes which factors are most influential, guiding priorities for intervention, while visually linking technical, institutional, and behavioral elements to illustrate the interplay between individual skills, workplace culture, and systemic support. Overall, this visualization provides a clear, intuitive map of actionable enablers and obstacles for STG adoption and complements the thematic analysis reported in the Results section. System-level barriers included delayed dissemination, stockouts, workload, and private sector influence, while audits, peer support, and digital tools provided opportunities.

**Table 2:** Barriers, facilitators, and COM-B aligned implementation adaptation of adherence to evidence-based standard antimicrobial treatment guidelines (STGs) among physicians in Ethiopia (*N* = 47).

Variable	Theme	Sub-theme/category	Frequency (*n*)	Percentage (%)
Barriers	Guideline related	Difficulty accessing and interpreting guidelines at the point of care	37	78.2
		Outdated content and lack of clinical depth	41	87.2
		Misalignment with patient preferences	32	68
	Physicians-related	Resistance from senior clinicians and faculty	28	59.6
		Inadequate training on STGs in medical education	40	85
		Low confidence in guideline accuracy or relevance	29	61.7
	Health system-related	Delays in disseminating updated guidelines	30	63.8
		Lack of on-the-job training and institutional advocacy	41	87.2
		Inaccessibility of guidelines	39	82.9
		Inaccessibility of essential antimicrobials	33	70.2
		Time constraints and clinical workload	38	80.8
		Influence of private health facility prescription practices	30	63.8
Facilitators		Perceptions of evidence-based antimicrobial prescription practices	29	61.7
		Institutional support and clinical audits	30	63.8
		Integration of STGs into daily clinical routines	33	70.2
		Inclusive guideline development process	27	57.4
		Peer collaboration and interdisciplinary support	26	55.3
		Positive perception of the STGs reliability	29	61.7
		Professional interest in and motivation to practice evidence-based healthcare	32	68
COM-B aligned implementation adaptation	Capability-focused	Training and capacity building on STGs use and app navigation	33	70.2
		Policy enforcement and educational integration	29	61.7
	Opportunity-focused	Digitalization of STGs through mobile applications	41	87.2
		Building infrastructure to support digital access	39	82.9
	Motivation-focused	Ensuring sustainability through policy, monitoring, and feedback	35	74.5

### Barriers to evidence-based STGs adherence structured using COM-B domains

Capability-related barriers

Guideline-related barriers

### Difficulty accessing and interpreting guidelines at the point of care

Difficulty accessing guidelines was reported by 37(78.2%) physicians (Table 2). They reported that current STGs are not user friendly, particularly during high clinical work load. The study participants highlighted challenges in locating relevant sections due to the lack of a table of contents and the cumbersome physical format.

*“The guidelines lack a table of contents*,* which makes it very difficult to quickly locate specific clinical information during patient care. When treating patients*,* I often find it challenging to access the relevant sections of the guideline because it is bulky and poorly organized.” (General practitioner and head of healthcare quality in a public general hospital)*.

### Outdated content and lack of clinical depth

41(87.2%) physicians expressed concerns that the STGs are not regularly updated and, therefore, may lag behind current scientific advancements and international standards (Table 2). This delay in revision results in significant gaps, particularly in specialized areas such as dermatology, cardiology, psychiatry, endocrinology, and emergency gynecology.

*“This Standard Treatment Guideline is not frequently updated*,* and as a result*,* it fails to incorporate the most recent scientific evidence and clinical recommendations. In medicine*,* where practices evolve rapidly*,* using outdated guidelines can lead to suboptimal or even inappropriate care.” (Pediatrician from a public general hospital)*.

41(87.2%) physicians reported that in the absence of locally relevant updates, they are often forced to rely on external or international references, potentially reducing adherence to national protocols.

*“Some sections of the STGs are extremely shallow and lack the clinical detail necessary for proper decision-making in real-world cases. In many instances*,* I have found inconsistencies between what the national STGs recommends and what international or specialty guidelines advise.” (General surgeon and inpatient department head at a public general hospital)*.

### Misalignment with patient preferences

One commonly reported barrier is the misalignment between patient expectations and clinical recommendations. 32(68%) physicians revealed that patients, especially those in public hospitals, demand injections or specific antibiotics with which they are familiar, regardless of whether they are medically necessary or guideline compliant (Table 2). This persistence is often influenced by prior experiences in private healthcare settings or cultural beliefs.

*“Patients often insist on receiving injections or specific medications they are familiar with*,* even if the Standard Treatment Guideline clearly advises against those choices. They see injections as more effective*,* and if you don’t comply*,* they may feel that they’re being denied proper treatment.” (Adult outpatient department head at a public primary hospital)*.

### Physician-related barriers

#### Resistance from senior clinicians and faculty

28(59.6%) senior staff members prefer using international guidelines, specialty textbooks, or personal clinical experience rather than the STGs approved by the Ministry of Health of Ethiopia and the Ethiopian Food and Drugs Authority (Table 2).

*“Senior physicians and faculty often don’t accept the Ethiopian Standard Treatment Guidelines and rarely refer to them in clinical decision-making. Instead*,* they encourage the use of textbooks or global guidelines*,* which may not align with our local context or available resources.” (General practitioner from a public referral hospital)*.

### Inadequate training on STGs in medical education

40(85%) early-career physicians reported receiving little or no formal training on the use of Ethiopia’s STGs (Table 2).

*“In medical school*,* we were never trained to use the STGs or even told how important they are for national clinical practice. Our education focused on international guidelines and reference books*,* which do not always reflect our local realities.” (ENT specialist from a public referral hospital)*.

#### Low confidence in guideline accuracy or relevance

29(61.7%) physicians expressed doubts about the accuracy and relevance of STGs, particularly for complex or multimorbid cases (Table 2).

*“There are times I don’t fully trust the STG*,* especially when it doesn’t cover complex clinical cases or when the content feels outdated compared with what we see in practice. In those situations*,* I feel compelled to consult other international guidelines or rely on my experience to make a confident treatment decision.” (General practitioner from a public general hospital)*.

Opportunity-related barriers

Health system-related barriers

### Delays in disseminating updated guidelines

30(63.8%) physicians expressed frustration regarding the slow and inconsistent distribution of updated STGs (Table 2).

*“Even when the STG is updated*,* it doesn’t always reach us on time. It would truly help if the updates were quickly shared in a digital format so all of us could easily access the latest recommendations.” (General practitioner from a public primary hospital)*.

### Lack of on-the-job training and institutional advocacy

41(87.2%) physicians rarely reported formal training, refresher courses, or feedback opportunities (Table 2).

*“I’ve never seen formal training*,* institutional advocacy or feedback sessions on the STG*,* even though it’s essential to our work. Without proper training and advocacy*,* it’s hard to ensure consistent and correct use across all healthcare settings.” (General practitioner at a public referral hospital)*.

### Inaccessibility of guidelines and essential antimicrobials

39(82.9%) physicians reported limited access to printed or digital STGs, and 33(70.2%) reported drug shortages (Table 2).

*“Hard copies are rarely available in OPDs or wards*,* and we may either have only one copy in our hospital CEO’s office.” (General practitioner at a public general hospital)*.

*“Some drugs recommended in the STG may not be available in our hospital. That was the challenge I faced*,* which made it hard to follow the guidelines strictly.” (General practitioner from a public general hospital)*.

### Time constraints and clinical workload

38(80.8%) physicians highlighted challenges of high patient load and time pressure (Table 2).

*“We often rely on our preexisting knowledge instead of the guidelines because referring to it takes considerable time*,* especially when we are dealing with a high patient load. Looking through the guideline while the patient is sitting in front of you may make them feel that you do not know what you are doing*,* even though you are trying to ensure evidence-based care. This perception affects patients’ trust and may impact their satisfaction with the service.” (Critical care physician at a public general hospital)*.

### Influence of private health facility prescription practices

33(70.2%) physicians reported patient expectations influenced by prior private care (Table 2).

*“Private hospitals routinely administer high-dose IV antibiotics and prescribe in ways that violate the STGs. When patients come to us*,* they expect the same irrational treatment*,* which makes strict adherence very difficult.” (General practitioner from a public general hospital)*.

### Facilitators of adherence to evidence-based STGs structured using COM-B domains

#### Capability-related facilitators

##### Perceptions of evidence-based antimicrobial prescription practices

Physicians generally recognize the importance of adhering to evidence-based STGs, with 29(61.7%) viewing them as tools for promoting rational drug use, preventing antimicrobial resistance, and enhancing patient outcomes (Table 2). Younger physicians, recently exposed to national stewardship messages, particularly highlighted their value.

*“I believe that the STG is a building block to our practice. This approach helps ensure consistency in prescribing and prevents guesswork. It should be available everywhere.” (General practitioner from a public general hospital)*.

Study participants emphasized the clarity, contextual relevance, and accessibility of the STGs, especially compared with bulkier international references. Local authorship and inclusion of essential medicines available in Ethiopian context strengthened physician trust.

*“Unlike large international textbooks*,* our STGs are concise and easier to grasp. They are developed by Ethiopian experts and directly reflect our local context.” (Head of critical care department at public general hospital)*.

#### Opportunity-related facilitators

##### Institutional support and clinical audits

Structured clinical audits focused on STG compliance were reported as key drivers of improved adherence. 30(63.8%) physicians noted that feedback and recognition from medical directors reinforced a culture of accountability and motivation (Table 2).

*“There is a clinical audit on adherence to clinical guidelines. At that time*,* everything was checked*,* and those who followed STG properly were encouraged and given feedback from medical directors. This encourages us to adhere to the STG.” (General practitioner from a public primary hospital)*.

### Integration of STGs into daily clinical routines

STG discussions integrated into routine clinical activities such as morning rounds, case presentations, and supervisory meetings helped normalize guideline use. 33(70.2%) physicians also made personal adjustments to improve accessibility, such as bookmarking or taking screenshots of frequently used pages (Table 2).

*“We have been discussing STGs in morning sessions*,* round sessions*,* at OPD*,* in clinical supervision*,* informing GPs and nurses to align to do according to STG. Doing so*,* we adhere to STG.” (General practitioner from a public general hospital)*.

### Inclusive guideline development process

Physicians involved in guideline development expressed stronger ownership and responsibility. 27(57.4%) reported that participation cultivated commitment, seeing guidelines as a product of their clinical realities (Table 2).

*“If you are part of the guideline development*,* you will feel that it is yours and use it more frequently.” (General practitioner from a public primary hospital)*.

### Peer collaboration and interdisciplinary support

26(55.3%) physicians reported that peer-to-peer learning and shared accountability aligned clinical decisions with the STG, particularly for complex antimicrobial cases (Table 2).

*“We share knowledge among senior/junior physicians. In morning sessions*,* seniors advise us to use STGs adherently. If a patient should be on first-line medication but has started a different one*,* with peer support*,* we adjust treatment according to guidelines*,* leading to good outcomes. We discuss AMR and receive support to follow guidelines.” (General practitioner from a public general hospital)*.

### Motivation-related facilitators

#### Positive perception of the STGs’ reliability

Physicians appreciated the clarity and simplicity of the STG. 29(61.7%) noted that locally relevant recommendations, concise format, and alignment with international standards enhanced credibility and usability (Table 2).

*“STGs are developed by Ethiopian physicians; they are straightforward and reliable.” (Critical care specialist from a public general hospital)*.

### Professional interest in and motivation to practice evidence-based healthcare

32(68%) physicians demonstrated strong personal commitment to using the STG as part of their professional identity (Table 2). Adherence was seen as ethical, rational, and aligned with evidence-based practice.

*“I believe that the Standard Treatment Guideline is a building block to our practice and it should be available everywhere*,* having it readily accessible in every ward and department would ensure that all professionals*,* regardless of experience level*,* can deliver evidence-based treatment. In my opinion*,* widespread availability is essential to foster a culture of rational prescribing and clinical accountability.” (General practitioner at a public general hospital)*.

Physicians emphasized that STGs serve as foundational tools, sharpening clinical decision-making and aligning healthcare with national protocols and international best practices.

### COM-B aligned implementation adaptation

#### Capability-focused implementation strategies

Physicians have proposed practical institutional and policy-level strategies to enhance adherence to STGs and evidence-based antimicrobial prescribing. Recommendations included digitalizing the STGs into user-friendly mobile applications with offline access, ensuring timely updates, and providing printed and electronic copies in clinical areas. Training interventions focused on integration into undergraduate and postgraduate curricula, structured on-the-job refreshers, and support for navigating digital tools. Strengthening institutional mechanisms such as clinical audits, feedback systems, and clinician involvement in guideline development was highlighted to foster ownership and accountability.

### Training and capacity building on STG use and app navigation

33(70.2%) physicians recommended a comprehensive training strategy that includes initial induction, refreshers, app navigation support, and feedback mechanisms (Table 2). Training was also seen as a platform for raising awareness of the purpose of guidelines, reinforcing their value, and standardizing clinical practice across facilities. The study participants stressed the importance of national-scale training rollouts supported by government logistics and incentives for participation.

*“Training on how to use the app and how to receive updates is essential*,* alongside other clinical training sessions.” (General practitioner from a public primary hospital)*.

### Policy enforcement and educational integration

29(61.7%) study participants suggested that undergraduate and postgraduate curricula should formally integrate STG training so that new clinicians are equipped with guideline literacy from the beginning (Table 2). Furthermore, regulatory enforcement, especially in private healthcare settings, was urged to address deviations from the national guidelines and mitigate antimicrobial misuse.

*“Medical students should be trained to use STGs starting from the undergraduate level to ensure long-term usage.” (Pediatric department head at a public referral hospital)*.

### Opportunity-focused implementation strategies

30(63.8%) physicians proposed institutional and policy-level strategies to enhance STG adherence and evidence-based antimicrobial prescribing. Key recommendations included digitalizing STGs into searchable mobile applications, timely updates, and accessible printed and electronic copies. Training focused on integration into medical curricula, on-the-job refreshers, and support for navigating digital tools. Strengthening institutional mechanisms such as audits, feedback, and clinician involvement was emphasized to foster ownership and accountability. System-level strategies included improving the drug supply chain, aligning patient expectations, enforcing private-sector policies, and cross-referencing with international guidelines. Collectively, these interventions address capability, opportunity, and motivation barriers, creating an enabling environment for sustained guideline adherence.

### Digitalization of STGs through mobile applications

41(87.2%) physicians cited the convenience, speed, and offline accessibility of app-based platforms as essential for real-time clinical decision-making (Table 2). Physicians compared their experiences with international digital tools such as UpToDate or Medscape, noting that having a national equivalent in app form would significantly increase adherence. The respondents envisioned features such as searchable databases, categorization by specialty or disease, and regular updates, all of which were embedded in a mobile interface tailored to their workflow.

*“It makes our life easy*,* as we can make right decisions at the right time. I would be very happy if it could be available as a mobile app.” (General practitioner from a public primary hospital)*.

*“Most physicians prefer using an app because it’s user friendly and can be accessed offline. This will benefit us by ensuring appropriate medication prescription.” (General practitioner from a public primary hospital)*.

### Building infrastructure to support digital access

39(82.9%) study participants highlighted the need for smartphones, reliable internet, workplace computers, technical support, and a dedicated team to ensure regular content updates (Table 2). For facilities with limited resources, access to desktop or tablet devices in clinical units was recommended to support STG access when mobile phones are unavailable or unusable.

*“We need strong financial and human resources to create sustainable access to standard treatment guidelines prepared in the form of mobile applications; this will ensure that regular updates reach us quickly.” (General practitioner from a public general hospital)*.

*“Some physicians might not have smartphones*,* or their devices might be dead. Having accessible computers at the workplace is essential.” (General practitioner from public primary hospital)*.

### Motivation-focused implementation strategies

At the system level, participants suggested improving the drug supply chain, aligning patient expectations through education campaigns, and enforcing policies in private healthcare settings. Cross-referencing with WHO and international guidelines was recommended to increase confidence in local STGs.

### Ensuring sustainability through policy, monitoring, and feedback

35(74.5%) study participants called for the integration of STG use into routine care pathways, linking them with health insurance reimbursement systems and emphasizing their role in preventing antimicrobial resistance (AMR) (Table 2). Regular feedback sessions, clinical audits, and supervision were also seen as central mechanisms for sustaining adherence.

*“When you’re involved in development of the standard treatment guideline*,* you feel ownership and use it more.” (General practitioner from a public teaching and referral hospital)*.

*“The MOH must monitor and regulate private facilities*,* which are sources of resistance due to STG violations.” (Medical director at a public general hospital)*.

## Discussion

This formative study provides insight into the complex challenges and enablers shaping physicians’ adherence to evidence-based antimicrobial treatment guidelines in Ethiopian public hospitals. By examining capability, opportunity, and motivation across diverse participants, the study identifies key determinants of prescribing practices and areas for targeted interventions. The findings integrate individual, institutional, and system-level factors, offering guidance for promoting rational antimicrobial use.

Several limitations should be noted. The qualitative design and purposive sampling may limit generalizability beyond the included public hospitals and regions. Male physicians predominated, reflecting staffing patterns, which may have influenced experiences reported. Private-sector practices were assessed mainly from public-sector physicians’ perspectives, as private practitioners were not directly interviewed.

In this study, many clinicians reported significant barriers related to guideline content and accessibility. In our study, physicians highlighted infrequent updates and limited clinical depth of the STGs as major concerns, which aligns with findings from other low- and middle-income settings^29,30^. Physicians in our study reported challenges locating information due to the bulkiness and poor indexing of the STGs, which aligns with observations from other LMICs where outdated or cumbersome guidelines reduced clinical utility^31^. The time-consuming nature of guideline consultation during patient care also contributes to low adherence, as busy clinicians prefer relying on their experience to maintain patient flow and confidence^32^.

Our findings indicate that limited exposure to STGs during medical training and low confidence in their applicability hinder guideline adherence, a pattern also observed in previous study^33^. The respondents proposed institutional and policy-level strategies to enhance STG adherence and evidence-based antimicrobial prescribing. Key recommendations included digitalizing STGs into searchable mobile applications, timely updates, and accessible digitally. Training focused on integration into medical curricula, on-the-job refreshers, and support for navigating digital tools. Strengthening institutional mechanisms such as audits, feedback, and clinician involvement was emphasized to foster ownership and accountability. System-level strategies included improving the drug supply chain, aligning patient expectations, enforcing private-sector policies, and cross-referencing with international guidelines. Collectively, these interventions address capability, opportunity, and motivation barriers, creating an enabling environment for sustained guideline adherence.

Using the COM-B framework helped to explain why some senior clinicians resist national guidelines. They may rely on entrenched clinical experience or international references (capability and motivation), perceive guideline use as conflicting with clinical workflow norms (opportunity), or seek to maintain authority over junior staff. Similarly, private health sector practices undermined adherence by establishing patient expectations for treatments not aligned with STGs, creating social and structural pressures on physicians working on public hospitals. This interpretation underscores that individual, institutional, and system-level factors interact to shape adherence, highlighting the need for multilevel strategies to foster capability, opportunity, and motivation simultaneously.

In this study, delayed dissemination, limited point-of-care access, and weak institutional advocacy were reported barriers, echoing challenges described in other settings^34^. Study participants noted that patients previously treated in private facilities often expected guideline-discordant therapies, a dynamic that complicates public-sector adherence and has been noted in similar contexts^35^. Private sector practice influences antimicrobial prescribing in public hospitals, as physicians reported that experiences from private health care facilities, including differing prescribing habits and limited use of STGs, shape routine clinical decision-making. The coexistence of public and private practice may contribute to variability in adherence when practices from private settings are carried into public hospitals, underscoring the need for better alignment of prescribing norms across sectors to support consistent evidence-based antimicrobial use. Our results align with findings from Kenya and Uganda, where limited guideline accessibility, frequent drug shortages, and inadequate training similarly impeded evidence-based prescribing^36,37^. In Ghana and Tanzania, senior clinicians’ reliance on international references and private-sector patient expectations similarly constrained evidence-based prescribing^38,39^. These parallels suggest that structural and social determinants of STG adherence are widespread across the region, reinforcing the relevance of institutional, digital, and policy-level strategies to strengthen guideline uptake.

Despite these barriers, several facilitators supported guideline adherence. Clinical audits, peer collaboration, and routine discussions reinforced evidence-based practice, while clinicians emphasized the value of digital access, such as mobile applications, to improve guideline usability and updates^40^. Comparable facilitators have also been observed in other sub-Saharan African countries. A study from Nigeria reported that making clinical guidelines available in user-friendly formats in soft copies and providing training on their use are associated with higher utilization of practice guidelines among physicians, suggesting that accessibility and capacity building support adherence^41^. The development of local antibiotic guideline packaged as a smartphone application was part of stewardship efforts that facilitated high adherence in medical wards, demonstrating the potential of digital tools to support guideline implementation in resource-limited settings^42^. In Rwanda, qualitative research on guideline used for surgical site infection prevention identified self-motivation, teamwork, peer support, and management support as key facilitators to using clinical standards, underscoring the role of organizational and social factors in enabling guideline uptake^43^.

Trust in locally adapted STGs and observed patient improvements further motivated adherence^44^. Stakeholders recommended policy and institutional interventions, including integrating STG training into medical curricula and strengthening digital infrastructure. Findings suggest that digital STGs can be realistically implemented by integrating them into routine ward activities, morning sessions, and supervisory meetings where clinicians discuss clinical cases and prescriptions. Similarly, participants’ positive experiences with clinical audits and feedback from medical directors indicate that embedding STG adherence indicators into existing audit and continuous professional development (CPD) programs could institutionalize evidence-based prescribing without creating parallel systems. These facilitators provide actionable entry points for enhancing adherence across public hospitals.

The COM-B framework helps explain the paradox of strong physician motivation coexisting with inconsistent adherence due to opportunity constraints, including limited guideline access, high workload, and private-sector pressures. These findings highlight the interplay of individual, institutional, and system-level factors. Addressing these collectively is essential to enhance rational antimicrobial use, mitigate resistance, and improve clinical outcomes. A targeted combination of capacity building, systemic support, and digital innovation is critical to sustaining evidence-based prescribing practices.

## Conclusion

Physicians in Ethiopian public hospitals face multiple barriers to adherence to standard treatment guidelines, including limited access to updated guidelines, gaps in training, systemic constraints, and patient and private health sector influences despite strong professional motivation. Key facilitators identified such as clinical audits, peer collaboration, and digital access to guidelines represents practical actions for improving adherence. These findings provide actionable insights for strengthening antimicrobial stewardship through targeted interventions, including mobile STG applications, structured training programs, and integration of guideline use into routine clinical and educational practices. While the study’s focus on selected public hospitals and predominance of male study participants may limit generalizability, the evidence offers clear foundation for policy, institutional reforms, and future research aimed at enhancing rational antimicrobial use and aligning private- and public-sector practices.

### Recommendations

Based on our findings, we recommend prioritizing both immediate and sustainable actions to improve adherence to evidence-based STGs. In short term, developing a smartphone application of the STGs with offline functionality and regular updates can enhance point-of-care decision-making for antimicrobial prescriptions and strengthen physicians’ capability. Integrating context-appropriate STGs adherence into continuous professional development (CPD) programs for hospital staff is essential to reinforce guideline use and standardize practice. In the long term, embedding STG training within undergraduate and postgraduate medical curricula will cultivate guideline literacy among new physicians, ensuring sustainable uptake of evidence-based practices. Future research should evaluate the effectiveness of digital STG tools, CPD-linked interventions, and cross sector stewardship strategies to inform policies and practices that support long term improvement in antimicrobial use.

### Strength and limitation of the study

This study used a rigorous qualitative design with diverse physician cadres across multiple public hospitals, underpinned by the COM-B and TDF frameworks, which enabled a systematic and theory-driven analysis of capability, opportunity, and motivation influencing guideline adherence. Robust data collection, thematic analysis, and triangulation across individual, institutional, and system levels enhanced the credibility and practical relevance of the findings. However, the predominance of male participants, reliance on public-sector perspectives to describe private-sector practices, and the focus on selected public hospitals limit transferability to other settings. These limitations highlight the need for future research involving private-sector clinicians and exploring potential gender-related differences in STGs adherence.

**Funding**.

**Human ethics and consent to participate**.

## Supplementary Information

Below is the link to the electronic supplementary material.

Supplementary Material 1

## Data Availability

The data are provided within the manuscript or supplementary information files.

## Abbreviations

AHRI: The Armauer Hansen Research Institute
AMR: Antimicrobial Resistance
AMS: Antimicrobial stewardship
COM-B: Capacity, Opportunity, and Motivation-Behavior
COREQ: Consolidated Criteria for Reporting Qualitative Research
CPD: Continuous Professional Development
IDI: In-depth interviews
IRB: Institutional Review Board
LMICs: Low- and middle-income countries
STGs: Standard treatment guidelines
TDF: Theoretical Domain Framework
WHO: The World Health Organization
