# Supplementary material for: Barriers to and facilitators of adherence to evidence-based standard antimicrobial treatment guidelines among physicians in Ethiopia: a formative qualitative study
Source: Sci Rep. 2026 Feb 27;16:11298. doi: 10.1038/s41598-026-41472-9 (PMC13048980; doi:10.1038/s41598-026-41472-9)

**Supplementary file 1: Data collection tool-interview guide**

| **Interviewer** |
| --- |
| **Date** |
| **Questions** |
| Name |
| Education status |
| Occupation/Department |
| Facility |
| Work Experience |
| Experience with guideline |
| Residence |
| Region |
| 1. **Do you find the guideline documents easy to read and understand? How does this affect your ability to follow them?** |
| *Probing question: Are there any specific sections of the STGs that you find especially complex or challenging to interpret?* |
| 1. **How well do you feel your medical training prepared you to follow antimicrobial guidelines (STGs) in your routine clinical practice?** |
| *Probing question: Are there specific areas of training you wish had focused more on guideline adherence?* |
| 1. **What challenges do you face in understanding or interpreting the antimicrobial treatment guidelines (STGs) in your daily clinical practice?** |
| *Probing question: Are there specific sections of the STGs that you find hard to apply or outdated information that impacts your clinical decisions?* |
| 1. **How confident are you in the evidence-based nature of the antimicrobial treatment guidelines (STGs), and does this affect your adherence?** |
| *Probing question: Can you describe a case where lack of confidence in the guidelines impacted your clinical approach?* |
| 1. **What are some of the challenges and opportunities in accessing up-to-date guidelines (STGs) at the point of care and has effect on your adherence to antimicrobial treatment recommendations?** |
| *Probing question: Can you describe a time when difficulty accessing current guidelines influenced your decision-making? Help respondent to mention in terms of Availability of UpToDate guideline, Training opportunities/challenges, Continuous support, feedback and leadership support* |
| 1. **How do time constraints and clinical workload pressures impact your ability to adhere to antimicrobial guidelines (STGs)?** |
| *Probing question: Can you provide an example where time limitations influenced a deviation from the guidelines?* |
| 1. **How do your colleagues and the broader medical community support your adherence to the guidelines (STGs)?** |
| *Probing question: Can you provide an example where peer support or recognition reinforced your commitment to following the guidelines?* |
| 1. **How accessible are the antimicrobial treatment guidelines (STGs) in your clinical practice, and how does this impact your ability to follow them?** |
| *Probing question: Can you describe a situation were having easy access to up-to-date guidelines positively influenced your adherence?* |
| 1. **How do individual patient needs influence your adherence to the guidelines (STGs)?** |
| *Probing question: Can you recall a case where patient-specific factors led you to adapt the guidelines to suit individual needs?* |
| 1. **Do you believe that having input into the development of antimicrobial treatment guidelines would enhance your adherence?** |
| *Probing question: How would having a role in guideline development improve your confidence in applying the guidelines?* |
| 1. **What specific steps or adjustments have you made in your clinical workflow to facilitate adherence to antimicrobial guidelines?** |
| *Probing question: Can you describe a particular change that has integrated guideline adherence more smoothly into your routine practice?* |
| 1. **How does confidence in having up-to-date knowledge of antimicrobial treatment guidelines (STGs) affect your adherence?** |
| *Probing question: How does knowing the guidelines are evidence-based influence your clinical decisions, particularly in complex cases?* |
| 1. **How would you feel about having a standard antibiotic treatment guideline of Ethiopia that you can easily access on your phone?** |
| *Probing question: What do you think is needed to successfully implement such a technology in your practice?* |
| *Probing question: What kind of infrastructure would support its use?* |
| *Probing question: What types of training do you think would be helpful?* |
| *Probing question: What factors do you believe are important for its long-term success?* |

**Supplementary file 2: Comprehensive codebook**

**Comprehensive codebook: Barriers and facilitators of adherence to evidence-based antimicrobial treatment guidelines among physicians in Ethiopia**

**Purpose:** This codebook provides a structured framework for analyzing qualitative data on the factors influencing physicians' adherence to Standard Treatment Guidelines (STGs) in Ethiopia. It is organized using the COM-B model (Capability, Opportunity, Motivation) to clearly link barriers and facilitators to potential intervention strategies.

**Legend:**

- **STG:** Standard Treatment Guideline
- **MOH:** Ministry of Health
- **GP:** General Practitioner
- **OPD:** Outpatient Department
- **AMR:** Antimicrobial Resistance

**I. Facilitators (enablers of STG adherence)**

| **COM-B dimension** | **Theme & definition** | **Code** | **Definition & significance** | **Representative quotes** |
| --- | --- | --- | --- | --- |
| **Motivation** | **Theme 1: Trust foundations** Confidence in the guidelines' scientific validity, local relevance, and institutional credibility | 1.1 Institutional credibility | Trust derived from the reputation of the developing institutions (MOH) and the expertise of the authors | "Because STGs are prepared by MOH with credible authors, I trust their evidence base" (GP from primary hospital) |
|  |  | 1.2 Observed efficacy | Witnessing positive patient outcomes when STGs are followed, reinforcing their practical value | "I see patients' outcome improve when using STGs." (GP from general hospital) "Witnessing patient improvement proves local guidelines applicability" (GP from referral hospital) |
|  |  | 1.3 Localization of global evidence | Trust built through the transparent adaptation of international evidence (e.g., WHO) to the local Ethiopian context | "They use WHO guidelines, research, and local context, no doubt about evidence quality" (GP from general hospital) "We use WHO guidelines but adjust for our drug formularies." (GP from general hospital) |
|  |  | 1.4 Trust through Co-creation | Enhanced credibility of guidelines when clinicians participate in evidence synthesis and recommendation drafting | "You trust what you helped create, we understand the evidence behind recommendations" (GP from referral hospital) "Having input makes me confident in the science and its applicability" (Infectious diseases specialist from referral hospital) |

| **Motivation** | **Theme 2: Stakeholder engagement in STG development**  Active involvement of physicians in guideline creation to foster ownership, context-relevance, and commitment | 2.1 Psychological ownership | The emotional investment and sense of responsibility clinicians develop when contributing to STG creation, leading to intrinsic motivation for adherence | "If you are part of the development, you feel it's yours and use it more frequently" (GP from general hospital) "If you have input in development, it impacts adherence, it's something you built" (GP from referral hospital) |
| --- | --- | --- | --- | --- |
|  |  | 2.2 Experiential integration | Incorporation of frontline clinical insights into STGs to ensure practical applicability to local patient populations and resource constraints | "I have eight years of clinical experience and can suggest management approaches for diverse conditions" (GP from referral hospital) "Specialists involved have field experience across Ethiopia" (GP from general hospital) |
|  |  | 2.3Inclusivity/engagement | Deliberate inclusion of providers from multiple specialties, care levels, and geographic settings to ensure comprehensive clinical coverage | "Taking inputs from physicians at all levels covers rare cases like pediatric dermatology" (Gynecologist from referral hospital) "We've asked for years to involve diverse perspectives of frontline clinicians" (Internist from referral hospital) |
|  |  | 2.4 Evidence-Context bridging | Transparent adaptation of international evidence to local epidemiology, drug availability, and resource constraints | "STGs are developed by Ethiopian physicians, they are straightforward and reliable" (Critical care specialist from general hospital) "Unlike large international textbooks, our STGs are concise and easier to grasp. They are developed by Ethiopian experts" (Critical care head from referral hospital) |
| **Opportunity** | **Theme 3: System-level enablers** Institutional structures, leadership, and policies that support guideline implementation | 3.1 Administrative Advocacy | Hospital leadership actively securing resources (e.g., guidelines, drugs) and championing STG use | "Hospitals can request guidelines from higher officials when missing" (GP from general hospital) |
|  |  | 3.2 Audit accountability | Systematic monitoring of prescribing practices with feedback loops and recognition for adherent prescribers | "There is clinical audit on adherence to STG; those clinicians who follow STG properly were encouraged and given feedback from medical directors" (GP from primary hospital) "Clinical audits identify adherent prescribers and so that directors give public recognition" (GP from general hospital) |
| **Opportunity**  **Opportunity** | **Theme 4: Workflow adaptations** Clinician-led innovations that integrate STGs into time-constrained clinical practice. | 4.1 Visual cognition aids | Transformation of text-based guidelines into flowcharts, wall charts, or color-coded posters for rapid information processing at the point of care | "We post STG algorithms for sepsis management in every ward" (GP from general hospital) "Color-coded antibiotic posters prevent dosing errors" (GP from general hospital) |
|  |  | 4.2 Digital micro-libraries | Personalized collections of STG excerpts (screenshots, bookmarks) on mobile devices for instant access to frequent clinical scenarios | "I screenshot frequent cases on my phone to avoid searching" (internist from referral hospital) "I select the most frequent case scenarios and screenshot the pages in STG, so I can adhere" (Obstetrics specialist from referral hospital) |
|  |  | 4.3 Protocol harmonization | Standardization of department-specific clinical pathways for high-burden conditions to enhance teamwork and reduce variation | "We created pneumonia bundles aligning nursing/medical protocols" (Infectious diseases specialist from referral hospital) |
|  | **Theme 5: Collegial support systems** Knowledge-sharing networks and interdisciplinary collaboration that enable collective problem-solving. | 5.1 Prescription stewardship | Peer-to-peer or pharmacist-led intervention to correct non-adherent prescriptions in a constructive, relationship-preserving manner | "When colleagues prescribe off-guideline, we discuss alternatives privately among ourselves" (GP from primary hospital) "I remember a clinical case in which a pharmacist intercepted conflicting prescription before dispensing" (GP from general hospital) |
|  |  | 5.2 Interdisciplinary clinical ward rounds | Structured case discussions across professional groups (physicians, pharmacists, nurses) to align care with STGs throughout the patient journey | "We share knowledge among senior/junior physicians, pharmacists, and nurses" (GP from general hospital) "Morning sessions and rounds with seniors reinforce AMR discussions and STG use" (GP from general hospital) |
|  |  | 5.3 Integration into clinical routine practice | Embedding STG discussion and use into regular hospital activities like morning meetings, rounds, and supervision | "We have been discussing STGs in morning sessions, round sessions, at OPD, in clinical supervisions in our hospital" (GP from general hospital) |
| **Capability** | **Theme 6: Guideline usability & clarity** Design features and content qualities that enhance understanding and practical application. | 6.1 Concise & contextual design | STGs are perceived as more straightforward and easier to grasp than large international textbooks, as they are tailored to the Ethiopian context | "Unlike large international textbooks, our STGs are concise and easier to grasp" (Critical care head from general hospital) "STGs are developed by Ethiopian physicians, they are straightforward and reliable" (Critical care specialist from referral hospital) |
|  |  | 6.2 Digital optimization | The availability of STGs in mobile-friendly digital formats (e.g., PDFs) that facilitate access outside of clinical stations | "PDFs on phones let us access guidelines anywhere." (GP from general hospital) |

**II. Barriers to STG adherence**

| **COM-B dimension** | **Theme & definition** | **Code** | **Definition & clinical impact** | **Representative quotes** |
| --- | --- | --- | --- | --- |
| **Opportunity** | **Theme 1: Private health facilities influence** Irrational prescribing practices in the private sector that undermine public sector adherence. | 1.1 Profit-driven prescribing | Systematic overuse of broad-spectrum antibiotics and injectables for financial gain and patient retention | "Private hospitals give IV ceftriaxone for colds - patients demand it from us" (GP from general hospital) "Private hospitals routinely administer high-dose IV antibiotics" (GP from general hospital) |
|  |  | 1.2 Therapeutic expectation transfer | Patients conditioned by private sector practices to expect specific (often irrational) treatments, creating pressure on public clinicians. | "Patients say 'The private doctor gave injections - why won't you?'" (GP from general hospital) "You want to follow the guideline, but when a patient comes in demanding what they got before" (OPD head from primary hospital) |
|  |  | 1.3 Resistance legacy & complex cases | Multi-drug-resistant infections originating from private sector overprescribing, rendering first-line STG recommendations ineffective | "We inherit ESBL infections from private clinics - forcing carbapenem use" (Surgeon from referral hospital) "I have seen patients who were overtreated, they are already resistant to treatments we give them" (Internal medicine specialist from referral hospital) |
|  |  | 1.4 Regulatory vacuum | The absence of effective monitoring and enforcement mechanisms for prescribing practices in private facilities | "No one monitors private health facilities." (Gynecologist from referral hospital) |
| **Opportunity** | **Theme 2: Physical-digital access Gaps** Systemic failures in ensuring the physical availability and digital usability of STGs. | 2.1 Physical scarcity of STG | Critical shortage of hard copies in clinical areas where they are needed most (wards, OPDs) | "The single STG copy is locked in the medical director's cabinet." (Infectious diseases specialist from referral hospital) "Hard copies are rarely available in OPDs or wards" (GP from primary hospital) |
|  |  | 2.2 Digital dysfunction | Unoptimized digital formats (e.g., large, non-searchable PDFs) that are impractical to use on small mobile screens during busy clinical encounters. | "Scrolling through 1200-page PDF on a 4-inch screen during trauma cases is impossible." (Clinical governance and quality department director from referral hospital) "Searching through numerous pages just to find a single medication... takes up valuable time." (Gynaecologist from referral hospital) |
|  |  | 2.3 Format limitations & poor organization | Non-user-friendly design elements such as tiny fonts, dense text, lack of spacing, and the absence of a table of contents. | "The guidelines lack a table of contents; it is a bulky document and poorly organized." (GP from general hospital) "Tiny fonts and dense text make reading exhausting." (Infectious diseases specialist from referral hospital) |
|  |  | 2.4 Delayed Dissemination | Significant time lags between the development of updated STGs and their distribution to frontline clinicians. | "Even when the STG is updated, it doesn't always reach us on time" (GP from primary hospital) |
| **Opportunity** | **Theme 3: Health system constraints** Broader systemic issues that directly impede the ability to follow STGs | 3.1 Essential drug stockouts | The frequent unavailability of STG-recommended medications in hospital formularies | "Some drugs recommended in the STG may not be available in our hospital and this made it hard to follow the guidelines" (GP from General hospital) |
|  |  | 3.2 Diagnostic limitations | Lack of essential diagnostic tools (e.g., culture tests), forcing physicians into empirical prescribing that may not align with STG assumptions | "Without culture tests, we guess STGs assume diagnostics we don't have" (GP from General hospital) |
| **Capability** | **Theme 4: Educational shortages** Insufficient pre-service and in-service training on STGs, compromising competence and confidence | 4.1 Pre-Service neglect | The absence of STG education and training within the medical school curriculum | "We learned zero about STGs in medical school only textbooks" (GP from primary hospital) "In medical school, we were not trained to use the local STG" (ENT specialist from referral hospital) |
|  |  | 4.2 In-Service absence | Lack of continuing professional development or refresher training on STG updates and use. | "Zero training on updates to STG, we rely on self-learning" (GP from general hospital) |
|  |  | 4.3 Pedagogical disconnect | Senior clinicians and faculty in teaching settings dismissing or discouraging the use of STGs in favor of international protocols or personal experience | "Faculty discourage STG use, instead they prefer international protocols" (GP from referral hospital) "Senior physicians and faculty often don’t accept the Ethiopian STG" (GP from referral hospital) |

| **Capability** | **Theme 5: Content deficiencies** Perceived limitations in the guideline content that reduce its clinical utility and reliability | 5.1 Specialty omissions & lack of depth | Inadequate coverage of key specialty areas (e.g., dermatology, psychiatry) and insufficient clinical detail for complex cases | "No protocols for dermatology, psychiatry, or neonatal care." (Infectious diseases specialist from referral hospital) "Some sections of the STG are extremely shallow and lack the clinical detail" (General surgeon from referral hospital) |
| --- | --- | --- | --- | --- |
|  |  | 5.2 Evidence-context misalignment | Recommendations that are perceived as outdated, contradict current international standards, or are irrelevant to the local context | "Diabetes doses contradict current international standards" (GP from referral hospital) "This Standard Treatment Guideline is not frequently updated" (Pediatrician from general hospital) |
|  |  | 5.3 Complexity gaps | Insufficient guidance for managing patients with multiple comorbidities, requiring time-consuming cross-referencing | "No advice for management of multimorbidity such as HIV with diabetes or hypertension cases, we waste time cross-referencing" (GP from general hospital) |

| **Opportunity** | **Theme 6: Workflow-context mismatch** Clinical realities and patient interactions that make adherence difficult in practice | 6.1 Time constraint | The impossibility of consulting lengthy guidelines during high-volume, fast-paced clinical settings | "With 150+ patients daily, I can't search 1800-page PDFs" (GP from general hospital) "We simply don’t have enough time to check the guideline while managing so many patients" (GP from general hospital) |
| --- | --- | --- | --- | --- |
|  |  | 6.2 Patient distrust & cultural pressure | Patients misinterpreting guideline consultation as a sign of incompetence or demanding specific treatments based on cultural preferences or prior private sector exposure | "Patients think I'm incompetent if I check guidelines during consults" (Quality head from general hospital) "Patients demand injections even when STGs prohibit them" (Clinical governance and quality department director from referral hospital) |
| **Motivation** | **Theme 7: Cultural & hierarchical resistance** Professional attitudes and social dynamics that undermine the authority and use of STGs | 7.1 Hierarchical dismissal | Senior clinicians openly mocking or rejecting STGs, creating a social environment where use is discouraged, especially among trainees | "Professors mock residents who use STGs, call them 'cookbook medicine" (Clinical governance and quality department director from general hospital) |
|  |  | 7.2 Low Trust in relevance | A perception that the STGs are outdated, incomplete, or unreliable for complex cases, reducing the intrinsic motivation to use them | "There are times I don’t fully trust the STG, especially when it doesn’t cover complex clinical cases" (GP from general hospital) |
|  |  | 7.3 Psychological Toll & ethical dilemmas | The internal conflict and frustration experienced when external pressures (patient demands, senior opinions) force clinicians to act against guideline recommendations and their professional judgment | "Patients preference due to their prior treatment in private clinics is frustrating because we see the harm, but they don’t" (Outpatient head at primary hospital) |

**III. Recommendations & implementation strategies**

| **COM-B dimension** | **Theme & definition** | **Code** | **Definition & proposed action** | **Representative quotes (Supporting the recommendation)** |
| --- | --- | --- | --- | --- |
| **Opportunity** | **Theme 1: Digital transformation** Developing mobile-first, intuitive solutions to overcome access and usability barriers | 1.1 Offline-accessible design | Creating STG applications that function fully without an internet connection to ensure reliability in low-connectivity settings | "Apps must work offline, many hospitals lack reliable data/internet connectivity" (Clinical governance and quality department director from referral hospital) |
|  |  | 1.2 Symptom-driven navigation | Designing an intuitive interface that allows clinicians to input symptoms and receive immediate, structured drug and management recommendations | "Let me input symptoms and get drug recommendations instantly" (GP from general hospital) |
|  |  | 1.3 Automated updates & searchability | Implementing push notifications for updates and robust search functions to eliminate revision lags and cumbersome navigation | "Push notifications when guidelines change, no more outdated PDFs" (Internal medicine specialist from referral hospital) "It makes our life easy; I would be very happy if it can be available as a mobile app" (GP from primary hospital) |
| **Capability** | **Theme 2: Educational reform** Restructuring training to bridge knowledge-practice gaps from undergraduate to continuing education | 2.1 Undergraduate integration | Embedding STG training and use directly into the medical school curriculum to ensure early exposure and familiarity | "Embed STGs in curricula so new graduates arrive familiar with it" (GP from general hospital) |
|  |  | 2.2 National trainer cadres & CME | Scaling quality instruction by recruiting and supporting trainers from all regions to deliver consistent Continuing Medical Education (CME) | "Recruit trainers from every region; cover their travel costs" (Clinical governance and quality department director) |
|  |  | 2.3 Just-in-time learning | Replacing theoretical workshops with contextualized, case-based coaching during clinical audits, rounds, and supervision | "Use clinical audit feedback sessions for case-based STG training, not theoretical workshops" (GP from primary hospital) |
| **Opportunity** | **Theme 3: Structural reinforcement** Strengthening policies, systems, and supply chains to create an enabling environment | 3.1 Accountability linkage | Incentivizing adherence by linking it to professional recognition, audit feedback, or potentially insurance reimbursement systems | "Link insurance payments to STG compliance" (GP from referral hospital); further emphasized (Implied: Promote motivation through recognition and awards*)* |
|  |  | 3.2 Private Sector regulation | Enforcing prescribing standards in private health facilities through MOH inspections and penalties for violators | "MOH must inspect private facilities and penalize violators." (GP from general hospital) |
|  |  | 3.3 Resource equity & drug supply | Ensuring universal access to both printed (pocket-sized) guidelines and a consistent supply of STG-recommended medications | "Print pocket-sized STGs for all departments including ophthalmology" (infectious diseases specialist from referral hospital) (Implied: strengthen drug supply chain) |
| **Motivation** | **Theme 4: Participatory stewardship** Fostering inclusive governance and community engagement to build trust and ownership | 4.1 Frontline co-design | Mandating the inclusion of practicing GPs and specialists from various levels of care in the STG development and revision process | "Include GPs in development, they know local disease patterns" (GP from general hospital) |
|  |  | 4.2 Community advocacy | Managing patient expectations through public education campaigns that explain the rationale behind STGs and address misconceptions | "Educate communities: using mobiles during clinical consultation is not neglect." (GP from referral hospital) (Implied: advocate for patient education campaigns) |
|  |  | 4.3 Strengthen professional motivation | Integrating STG adherence into mentorship and supervision programs, and promoting cross-referencing with international guidelines to build confidence | Integrate STG use into supervision, promote cross-referencing with WHO guidelines (GP from general hospital) |

**Supplementary file 3:** **The Consolidated Criteria for Reporting Qualitative Research (COREQ) 32-item checklist**

| **No. Item** | **Guide questions/description** | **Reported on page #** |
| --- | --- | --- |
| **Domain 1: Research team and reﬂexivity** |  |  |
| *Personal Characteristics* |  |  |
| 1. Inter viewer/facilitator | Which author/s conducted the interview or focus group? | 8 |
| 2. Credentials | What were the researcher’s credentials? E.g. PhD, MD | 8 |
| 3. Occupation | What was their occupation at the time of the study? | 9 |
| 4. Gender | Was the researcher male or female? | 9 |
| 5. Experience and training | What experience or training did the researcher have? | 8 & 9 |
| *Relationship with participants* |  |  |
| 6. Relationship established | Was a relationship established prior to study commencement? | **9 -11** |
| 7. Participant knowledge of the interviewer | What did the participants know about the researcher? e.g. personal goals, reasons for doing the research | **9 -11** |
| 8. Interviewer characteristics | What characteristics were reported about the inter viewer/facilitator? e.g. Bias, assumptions, reasons and interests in the research topic | **9 -11** |

| **Domain 2: study design** |  |  |
| --- | --- | --- |
| *Theoretical framework* |  |  |
| 9. Methodological orientation and Theory | What methodological orientation was stated to underpin the study? e.g. grounded theory, discourse analysis, ethnography, phenomenology, content analysis | 5 |
| *Participant selection* |  |  |
| 10. Sampling | How were participants selected? e.g. purposive, convenience, consecutive, snowball | 6 |
| 11. Method of approach | How were participants approached? e.g. face-to-face, telephone, mail, email | 7 |
| 12. Sample size | How many participants were in the study? | 8 |
| 13. Non-participation | How many people refused to participate or dropped out? Reasons? | 9 |
| *Setting* |  |  |
| 14. Setting of data collection | Where was the data collected? e.g. home, clinic, workplace | 4 |
| 15. Presence of non-participants | Was anyone else present besides the participants and researchers? | 5 |
| 16. Description of sample | What are the important characteristics of the sample? e.g. demographic data, date | 5 |
| *Data collection* |  |  |
| 17. Interview guide | Were questions, prompts, guides provided by the authors? Was it pilot tested? | 8 |
| 18. Repeat interviews | Were repeat inter views carried out? If yes, how many? | **9** |
| 19. Audio/visual recording | Did the research use audio or visual recording to collect the data? | 9&10 |
| 20. Field notes | Were ﬁeld notes made during and/or after the interview or focus group? | 9&10 |
| 21. Duration | What was the duration of the inter views or focus group? | 9&10 |
| 22. Data saturation | Was data saturation discussed? | 9&10 |
| 23. Transcripts returned | Were transcripts returned to participants for comment and/or correction? | 9&10 |
| **Domain 3: analysis and ﬁndings** |  |  |
| *Data analysis* |  | 11&12 |
| 24. Number of data coders | How many data coders coded the data? | 13 |
| 25. Description of the coding tree | Did authors provide a description of the coding tree? | 13_14 |
| 26. Derivation of themes | Were themes identiﬁed in advance or derived from the data? | 11-14 |
| 27. Software | What software, if applicable, was used to manage the data? | **10&11** |
| 28. Participant checking | Did participants provide feedback on the ﬁndings? | **15** |
| *Reporting* |  |  |
| 29. Quotations presented | Were participant quotations presented to illustrate the themes/ﬁndings? Was each quotation identiﬁed? e.g. participant number | 15-21 |
| 30. Data and ﬁndings consistent | Was there consistency between the data presented and the ﬁndings? | **22-24** |
| 31. Clarity of major themes | Were major themes clearly presented in the ﬁndings? | **24-26** |
| 32. Clarity of minor themes | Is there a description of diverse cases or discussion of minor themes? | **27** |

**Supplementary file 4: Ethical approval letters**


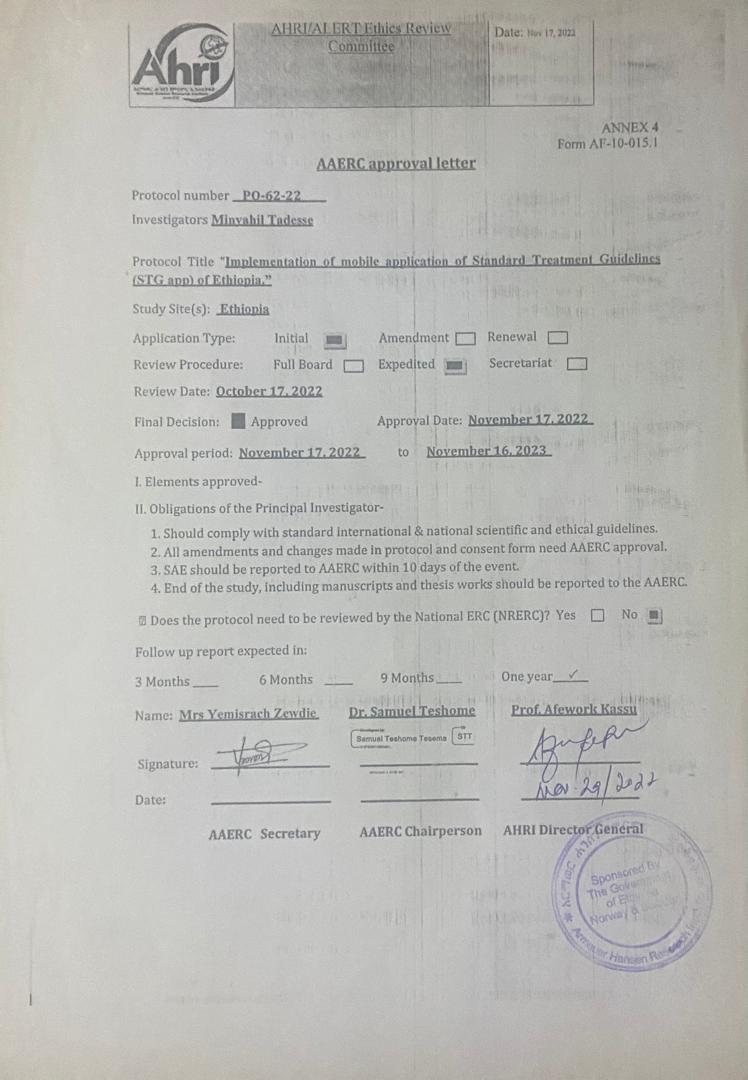


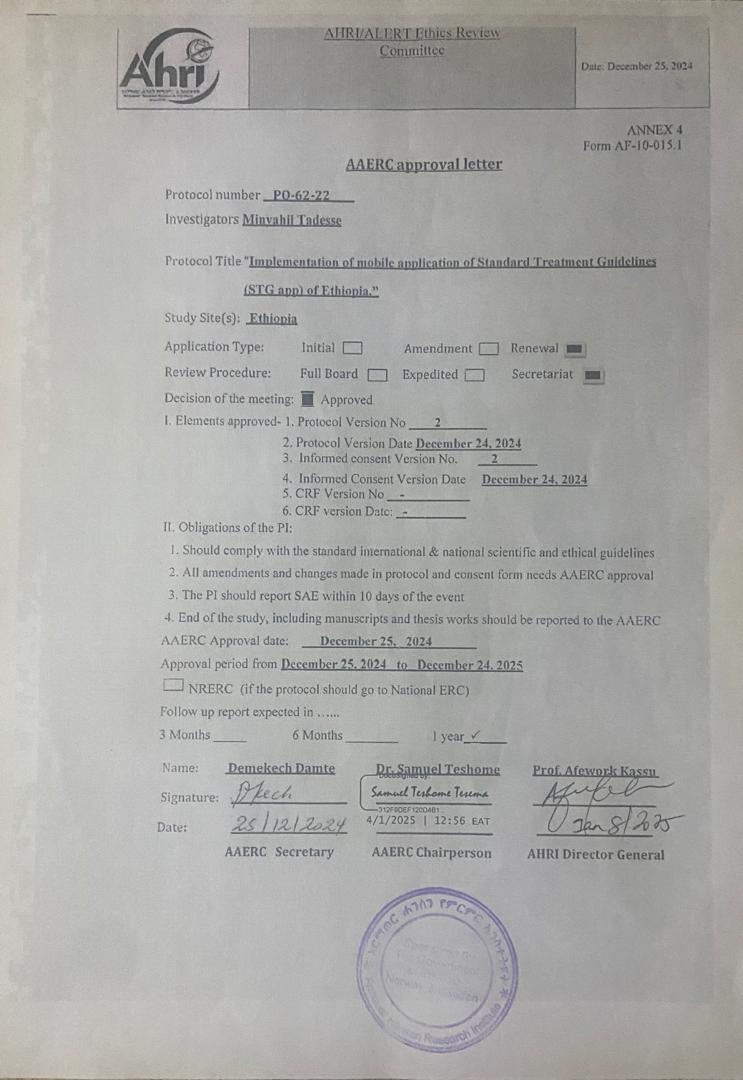


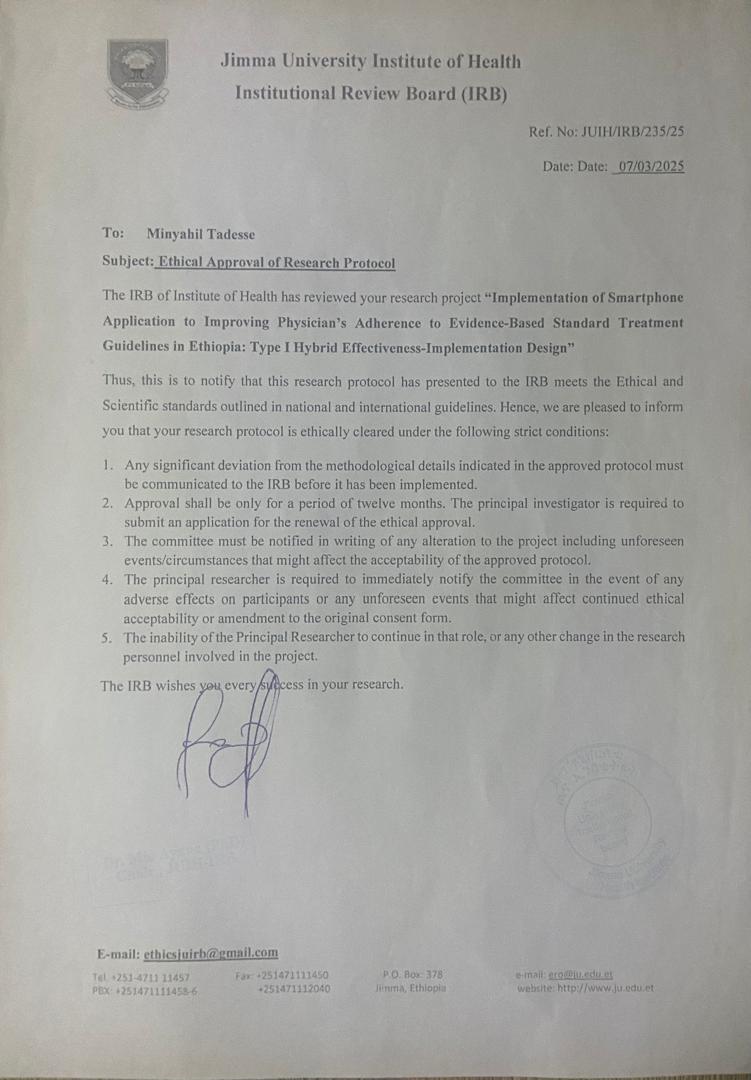


**Supplementary file 5: Findings mapped to the COM-B framework**

| **COM-B framework** | **Barriers (themes, subthemes, quotes)** | **Facilitators (themes, subthemes, quotes)** | **Implementation strategies to address barriers** |
| --- | --- | --- | --- |
| **Capability (Knowledge, skills, confidence to use STGs)** | **1. Inadequate training & guideline literacy** • Little exposure in medical school, emphasis on international textbooks “In medical school, we were not trained to use the local STG; our education focused on international guidelines” (ENT specialist from referral hospital)  **2. Difficulty accessing & interpreting guidelines** • Bulky format, lack of table of contents, not user-friendly in busy encounters “The guidelines lack a table of contents; it is a bulky document and poorly organized” (GP from general hospital) “Searching through numerous pages just to find a single medication or recommendation takes up valuable time” (Gynecologist from referral hospital)  **3. Outdated content & lack of clinical depth** • Not regularly updated, shallow in complex specialties “This Standard Treatment Guideline is not frequently updated and using outdated guidelines can lead to inappropriate care” (Pediatrician from general hospital) “Some sections of the STG are extremely shallow and lack the clinical detail necessary for proper decision-making” (General surgeon and inpatient department head)  **4. Low confidence in accuracy/relevance** • Perception of outdated or incomplete information, especially for complex cases “There are times I don’t fully trust the STG, especially when it doesn’t cover complex clinical cases” (GP from general hospital) | **1. Clarity and local authorship** • Concise, contextual, aligned with Ethiopian realities “Unlike large international textbooks, our STGs are concise and easier to grasp. They are developed by Ethiopian experts and directly reflect our local context” (Critical care head) “STGs are developed by Ethiopian physicians, they are straightforward and reliable” (Critical care specialist from general hospital)  **2. Personal strategies for access** • Individual workarounds to overcome usability issues. “I select the most frequent case scenarios and screenshot the pages in STG, so I can adhere to the guideline all the time without wasting time to search” (Obstetrics specialist from referral hospital) | • Digitalization of STGs through searchable mobile applications with offline access • Integrate STG training into undergraduate and postgraduate curricula • Regular revision and update of content with clinical depth • Structured training on STG use, refreshers, and app navigation |
| **Opportunity (External environment, resources, system enablers)** | **1. Health system constraints** • Essential drug shortages, delayed dissemination of updates, lack of printed/digital access “Some drugs recommended in the STG may not be available in our hospital and this made it hard to follow the guidelines.” (GP from General hospital) “Even when the STG is updated, it doesn’t always reach us on time... Hard copies are rarely available in OPDs or wards” (GP from primary hospital)  **2. Workload & time pressure** • Fast-paced settings discourage consultation “We simply don’t have enough time to check the guideline while managing so many patients” (GP from general hospital)  **3. Private health facilities influence** • Irrational prescribing undermines consistency and shapes patient expectations “Private hospitals routinely administer high-dose IV antibiotics and patients coming to our hospital with prior treatment from private hospitals expect the same irrational treatment.” (GP from general hospital)  **4. Resistance from senior clinicians** • Reliance on textbooks and international guidelines “Senior physicians and faculty often don’t accept the Ethiopian Standard Treatment Guidelines and rarely refer to them during point-of-care decision making” (GP from referral hospital) | **1. Institutional support & clinical audits** • Accountability and feedback improve adherence “There is clinical audit on adherence to STG; those clinicians who follow STG properly were encouraged and given feedback from medical directors.” (GP from primary hospital)  **2. Peer collaboration & interdisciplinary support** • Knowledge sharing across cadres and seniority levels “We share knowledge among senior/junior physicians, pharmacists, and nurses. In addition, seniors advise us to use STGs adherently” (GP from general hospital)  **3. Inclusive guideline development** • Ownership encourages adherence. “If you are part of the guideline development, you will feel that it is yours and use it more frequently.” (GP from primary hospital)  **4. Integration into clinical routines** • Embedding STGs into daily workflows “We have been discussing STGs in morning sessions, round sessions, at OPD, in clinical supervisions in our hospital” (GP from general hospital) | • Digital dissemination system with mobile app and searchable formats. • Ensure availability of printed and electronic copies in wards and OPDs • Strengthen drug supply chain to ensure STG-recommended drugs are available • Institutionalize audits, feedback, and supervisory visits • Advocate for patient education campaigns to align expectations • Policy enforcement to regulate private sector prescribing |
| **Motivation (Beliefs, values, professional identity)** | **1. Pressure from patients & seniors** • Social and cultural influences override professional intentions “You want to follow the guideline, but when a patient comes in demanding what they got before in private health facilities, they really pose pressure” (OPD head, primary hospital)  **2. Psychological toll & ethical dilemmas** • Conflict between guideline adherence and external pressure “Patients preference due to their prior treatment in private clinics is frustrating because we see the harm, but they don’t” (Outpatient head at primary hospital)  **3. Low trust in STG relevance:** • Outdated or shallow guidelines reduce motivation to use them | **1. Professional Identity & Ethical Duty** • STGs seen as a cornerstone of evidence-based practice and integrity “As someone who advocate about rational drug use, I see the STG as more than a document, it reflects evidence-based recommendations I personally stand for” (GP from general hospital)  **2. Positive Perceptions & Trust** • Trust in MOH and Ethiopian experts “STGs are developed by Ethiopian physicians, they are straightforward and reliable” (Critical care specialist from general hospital)  **3. Positive experiences & confidence boost** • STGs provide structured support. “The STG provides structured guidance that boosts my confidence during clinical decision-making” (GP from primary hospital)  **4. Motivation to innovate (digital solutions)** • Desire for tools that enable adherence “It makes our life easy as we can make right clinical decisions at the right time; I would be very happy if it can be available as a mobile app” (GP from primary hospital)  **5. Personal narratives of harm** • Witnessing negative outcomes reinforces the urgency of rational use “I have seen patients who were overtreated with unnecessary antibiotics from private clinics; by the time they come to us, they are already resistant to treatments we give them” (Internal medicine specialist) | • Promote professional motivation through recognition, awards, and audit feedback • Encourage clinician involvement in guideline development to enhance ownership • Integrate STG use into supervision and mentorship programs • Promote cross-referencing with WHO/international guidelines to strengthen confidence • Raise awareness on AMR and harms of irrational use through CME • Expedite development of a digital STG app to meet expressed desire |

**Supplementary file 6: COM-B analysis of the barriers and facilitators to evidence-based standard treatment guideline (STG) adherence in Ethiopia**

| **COM-B dimensions** | **Barriers (themes, subthemes, quotes)** | **Facilitators (themes, subthemes, quotes)** | **Implementation strategies to address barriers** |
| --- | --- | --- | --- |
| **Capability** *(Knowledge, skills, confidence to use STGs)* | **1. Inadequate training & guideline literacy:** • Little exposure in medical school, emphasis on international textbooks. *“In medical school, we were not trained to use the local STG; our education focused on international guidelines.” (ENT specialist from referral hospital)*  **2. Difficulty accessing & interpreting guidelines:** • Bulky format, lack of table of contents, not user-friendly in busy encounters. *“The guidelines lack a table of contents; it is a bulky document and poorly organized.” (GP from general hospital)* *“Searching through numerous pages just to find a single medication or recommendation takes up valuable time.” (Gynaecologist from referral hospital)*  **3. Outdated content & lack of clinical depth:** • Not regularly updated, shallow in complex specialties. *“This Standard Treatment Guideline is not frequently updated and using outdated guidelines can lead to inappropriate care.” (Pediatrician from general hospital)* *“Some sections of the STG are extremely shallow and lack the clinical detail necessary for proper decision-making…” (General surgeon and inpatient department head)*  **4. Low confidence in accuracy/relevance:** • Perception of outdated or incomplete information, especially for complex cases. *“There are times I don’t fully trust the STG, especially when it doesn’t cover complex clinical cases” (GP from general hospital)* | **1. Clarity and local authorship:** • Concise, contextual, aligned with Ethiopian realities. *“Unlike large international textbooks, our STGs are concise and easier to grasp. They are developed by Ethiopian experts and directly reflect our local context.” (Critical care head)* *“STGs are developed by Ethiopian physicians, they are straightforward and reliable.” (Critical care specialist from general hospital)*  **2. Personal strategies for access:** • Individual workarounds to overcome usability issues. *“I select the most frequent case scenarios and screenshot the pages in STG, so I can adhere to the guideline all the time without wasting time to search.” (Obstetrics specialist from referral hospital)* | • **Digitalization of STGs** through searchable mobile applications with offline access. • **Integrate STG training** into undergraduate and postgraduate curricula. • **Regular revision and update** of content with clinical depth. • **Structured training** on STG use, refreshers, and app navigation. |
| **Opportunity** *(External environment, resources, system enablers)* | **1. Health system constraints:** • Essential drug shortages, delayed dissemination of updates, lack of printed/digital access. *“Some drugs recommended in the STG may not be available in our hospital and this made it hard to follow the guidelines.” (GP from General hospital)* *“Even when the STG is updated, it doesn’t always reach us on time... Hard copies are rarely available in OPDs or wards” (GP from primary hospital)*  **2. Workload & time pressure:** • Fast-paced settings discourage consultation. *“We simply don’t have enough time to check the guideline while managing so many patients.” (GP from general hospital)*  **3. Private health facilities influence:** • Irrational prescribing undermines consistency and shapes patient expectations. *“Private hospitals routinely administer high-dose IV antibiotics and patients coming to our hospital with prior treatment from private hospitals expect the same irrational treatment.” (GP from general hospital)*  **4. Resistance from senior clinicians:** • Reliance on textbooks and international guidelines. *“Senior physicians and faculty often don’t accept the Ethiopian Standard Treatment Guidelines and rarely refer to them during point-of-care decision making” (GP from referral hospital)* | **1. Institutional support & clinical audits:** • Accountability and feedback improve adherence. *“There is clinical audit on adherence to STG; those clinicians who follow STG properly were encouraged and given feedback from medical directors.” (GP from primary hospital)*  **2. Peer collaboration & interdisciplinary support:** • Knowledge sharing across cadres and seniority levels. *“We share knowledge among senior/junior physicians, pharmacists, and nurses. In addition, seniors advise us to use STGs adherently.” (GP from general hospital)*  **3. Inclusive guideline development:** • Ownership encourages adherence. *“If you are part of the guideline development, you will feel that it is yours and use it more frequently.” (GP from primary hospital)*  **4. Integration into clinical routines:** • Embedding STGs into daily workflows. *“We have been discussing STGs in morning sessions, round sessions, at OPD, in clinical supervisions in our hospital” (GP from general hospital)* | • **Digital dissemination system** with mobile app and searchable formats. • **Ensure availability** of printed and electronic copies in wards and OPDs. • **Strengthen drug supply chain** to ensure STG-recommended drugs are available. • **Institutionalize audits, feedback, and supervisory visits.** • **Advocate for patient education campaigns** to align expectations. • **Policy enforcement** to regulate private sector prescribing. |
| **Motivation** *(Beliefs, values, professional identity)* | **1. Pressure from patients & seniors:** • Social and cultural influences override professional intentions. *“You want to follow the guideline, but when a patient comes in demanding what they got before in private health facilities, they really pose pressure.” (OPD head, primary hospital)*  **2. Psychological toll & ethical dilemmas:** • Conflict between guideline adherence and external pressure. *“Patients preference due to their prior treatment in private clinics is frustrating because we see the harm, but they don’t.” (Outpatient head at primary hospital)*  **3. Low trust in STG relevance:** • Outdated or shallow guidelines reduce motivation to use them. | **1. Professional Identity & Ethical Duty:** • STGs seen as a cornerstone of evidence-based practice and integrity. *“As someone who advocate about rational drug use, I see the STG as more than a document, it reflects evidence-based recommendations I personally stand for.” (GP from general hospital)*  **2. Positive Perceptions & Trust:** • Trust in MOH and Ethiopian experts. *“STGs are developed by Ethiopian physicians, they are straightforward and reliable.” (Critical care specialist from general hospital)*  **3. Positive experiences & confidence boost:** • STGs provide structured support. *“The STG provides structured guidance that boosts my confidence during clinical decision-making.” (GP from primary hospital)*  **4. Motivation to innovate (digital solutions):** • Desire for tools that enable adherence *“It makes our life easy as we can make right clinical decisions at the right time. I would be very happy if it can be available as a mobile app.” (GP from primary hospital)*  **5. Personal narratives of harm:** • Witnessing negative outcomes reinforces the urgency of rational use *“I have seen patients who were overtreated with unnecessary antibiotics from private clinics; by the time they come to us, they are already resistant to treatments we give them” (Internal medicine specialist)* | • **Promote professional motivation** through recognition, awards, and audit feedback. • **Encourage clinician involvement** in guideline development to enhance ownership. • **Integrate STG use** into supervision and mentorship programs. • **Promote cross-referencing** with WHO/international guidelines to strengthen confidence. • **Raise awareness on AMR** and harms of irrational use through CME. • **Expedite development of a digital STG app** to meet expressed desire. |

**Supplementary file 7: Source of fund**
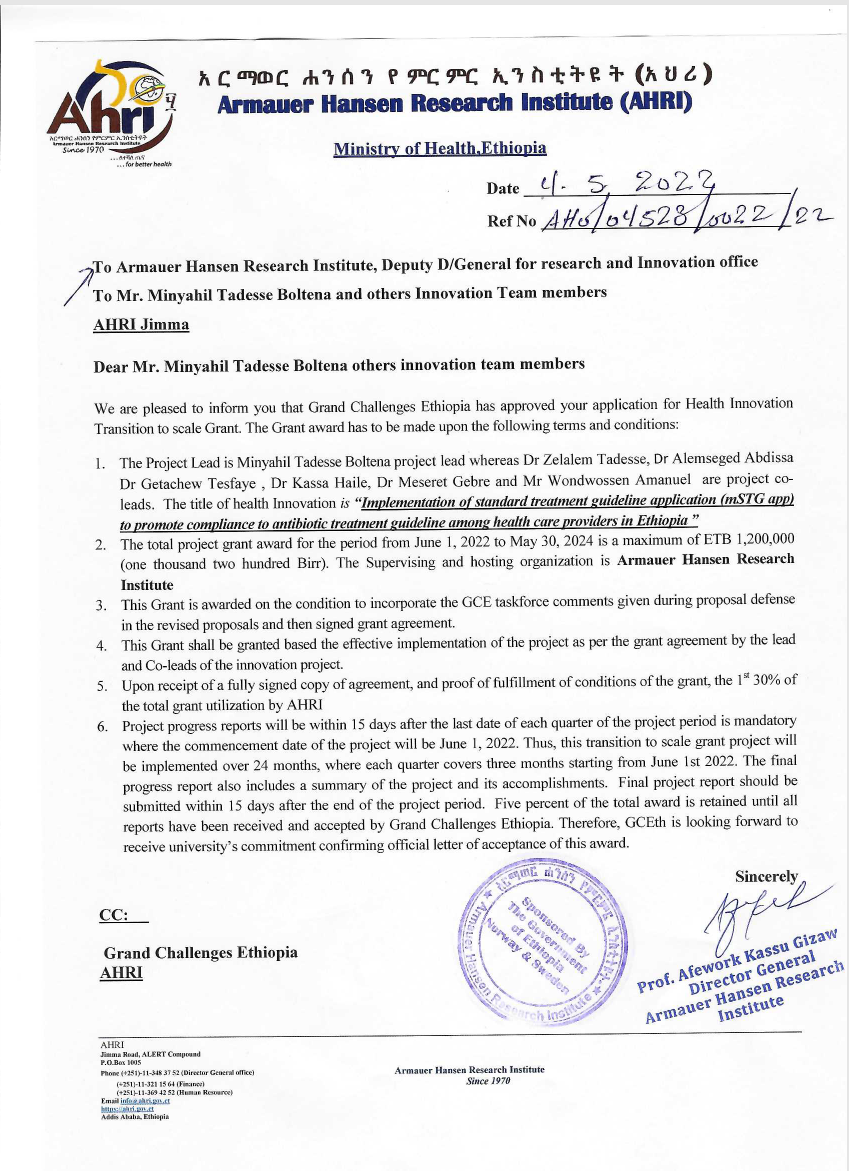

Supplement: Supplementary file 1 — Supplementary Material 1 [file 41598_2026_41472_MOESM1_ESM.docx]
